# Time is of the Essence: A Review of Electroencephalography (EEG) and Event-Related Brain Potentials (ERPs) in Language Research

**DOI:** 10.1007/s10484-017-9371-3

**Published:** 2017-07-11

**Authors:** Anna M. Beres

**Affiliations:** 1 Department of Cognitive Neuroscience and Neuroergonomics, Faculty of Management and Social Communication, Institute of Applied Psychology, Jagiellonian University, ul. Łojasiewicza 4, 30-348 Kraków, Poland; 2 Institute of Psychology, SWPS University of Social Sciences and Humanities, ul. Chodakowska 19/31, 03-815 Warsaw, Poland

**Keywords:** EEG, ERPs, Electroencephalography, Event-related brain potentials, Language

## Abstract

The discovery of electroencephalography (EEG) over a century ago has changed the way we understand brain structure and function, in terms of both clinical and research applications. This paper starts with a short description of EEG and then focuses on the event-related brain potentials (ERPs), and their use in experimental settings. It describes the typical set-up of an ERP experiment. A description of a number of ERP components typically involved in language research is presented. Finally, the advantages and disadvantages of using ERPs in language research are discussed. EEG has an extensive use in today’s world, including medical, psychology, or linguistic research. The excellent temporal resolution of EEG information allows one to track a brain response in milliseconds and therefore makes it uniquely suited to research concerning language processing.

## Introduction

Electroencephalography(EEG) has come a long way since its discovery 140 years ago by an English physician Richard Caton. In 1875, he obtained the first EEG from open brains of monkeys and rabbits. Almost 50 years later, in 1924, Hans Berger made the first EEG recording on the human scalp, by using simple radio equipment in order to amplify the electrical activity of the brain, and obtained a written output on paper. He claimed that brain activity that is observed through the use of EEG can change in a consistent, reliable and recognizable fashion when the state of the patient changes, such as going from relaxation to alertness, sleep, lack of oxygen (Bronzino 1995). This breakthrough gave rise for the research of the years to come and the varied applications of EEG use today.

The average human brain has about 86 billion neurons (Herculano-Houzel 2009), and the communication between them is the key brain activity. They are excitable cells with intrinsic electrical properties, and their activity results in magnetic as well as electrical fields, which can then be recorded with the use of recording electrodes. The EEG is the recording of the summed electrical activity of populations of neurons called pyramidal cells, measured with the use of electrodes placed on the scalp and graphed over time. It is an alternating current that fluctuates from positive to negative depending on a number of factors, including changes in the permeability of the cell membrane that are induced by excitatory or inhibitory inputs from other neurons. There are two main types of neuronal activity: action potentials and postsynaptic potentials. Action potentials are the result of the very rapid depolarization of a neuron mediated mainly by changes in permeability of the membrane to sodium and potassium ions. They occur when the cell depolarizes to a certain degree from its negative resting state potential. Once that threshold is reached, there is a rapid firing of the action potential (about 1 ms.) from the beginning of the axon at the cell body down to the axon terminals. Postsynaptic potentials are mediated by a number of neurotransmitter systems and, as a result of synaptic activation, generally entail slower changes in membrane potentials (Lopes da Silva 2010). They are voltages produced when the neurotransmitters bind to the receptors on the membrane of the postsynaptic cell, making ion channels open or close. Reliably, EEG can only record postsynaptic potentials. Due to action potentials being very rapid and brief, in addition to having to travel down the axon at a fixed rate, the electrodes placed on the scalp simply cannot detect them. Postsynaptic potentials, on the other hand, represent the change in electrical charge outside the membrane and this lasts in the extra-cellular space for up to 200 ms. The extra-cellular electrical charge, positive or negative, is what is measured with electrodes placed on the scalp. Pyramidal cells are like little batteries in that they have polarity—if one end of the dendrite is positive, the other is negative. Whether the charge outside the dendrite at the top of a pyramidal cell is positive or negative depends on two factors; first, whether an inhibitory or excitatory stimulus has come to the synaptic junction from the axon of another cell and, second, whether that synapse is proximal or distal to the cell body. For example, if an excitatory stimulus comes in near the distal end of the dendrite (near the surface of the cortex) the change in permeability of the membrane allows Na^+^ to rush into the cell at that point leaving the extracellular space negative (since the pyramidal cell acts like a battery, the extra-cellular space at the proximal end of the dendrite will be positive). The EEG electrode on the scalp will record a negative extracellular potential if the same thing is happening at the same time to a large number of pyramidal cells in the same macro-column of cells that lies below the electrode. The EEG thus represents the algebraic sum of excitatory and inhibitory postsynaptic potentials.

EEG plays a crucial role in many aspects of today’s research. It is used in medicine, where monitoring brain activity (or the lack thereof) is useful in determining brain death in patients, areas of damage following a stroke or head trauma, epileptic activity, sleep disorders, and many others. In other research, it is useful in investigating various cognitive functions, such as memory or attention; it is also used in language and clinical research; for example, studies that investigate EEG patterns in individuals with aphasia.

## Event-Related Brain Potentials (ERPs)

For decades, EEG recording was of great use in research and clinical settings. However, it is very difficult, if not impossible, to use the raw, continuous recording to examine the specific neural activity as a function of certain cognitive processes.

Event-related brain potentials are small parts of the continuous EEG recording, which are evoked in response to stimuli, such as viewing of pictures or words on the computer screen. In cognitive neuroscience experiments, it is not very informative to just use a continuous EEG recording. If we are interested in, for example, how the brain deals with language comprehension or production, we need the recording to reflect the modulation of brain activity by that particular task, in a precise moment in time. Therefore, it is necessary to look at the ERPs, rather than a continuous EEG recording. ERPs are obtained by time-locking the stimuli so that we know at exactly which point in time the stimuli were presented and then we analyze the brain response to a particular stimulus, such as a sound, word, picture, and so on.

ERPs are used in a range of psychological experiments that aim to investigate various aspects of cognitive processes, such as language comprehension and production, memory, attention, amongst many others. ERPs cannot be typically seen within the raw EEG recording, because of their very small amplitude (Teplan 2002). Therefore, they need to be singled out from the continuous recording by creating an average of recording periods, known as epochs, which are time-locked to repeated presentations of the same stimulus. The spontaneous EEG fluctuations, unrelated to stimulus presentation, are averaged out, resulting in the ERP wave, which reflects only the activity persistently related to the time-locked presentation of stimuli. Therefore, it can be said that the ERPs mirror the neuronal activity evoked by the repeated presentation of a stimulus.

### How Does it Work?

**Fig. 1:**
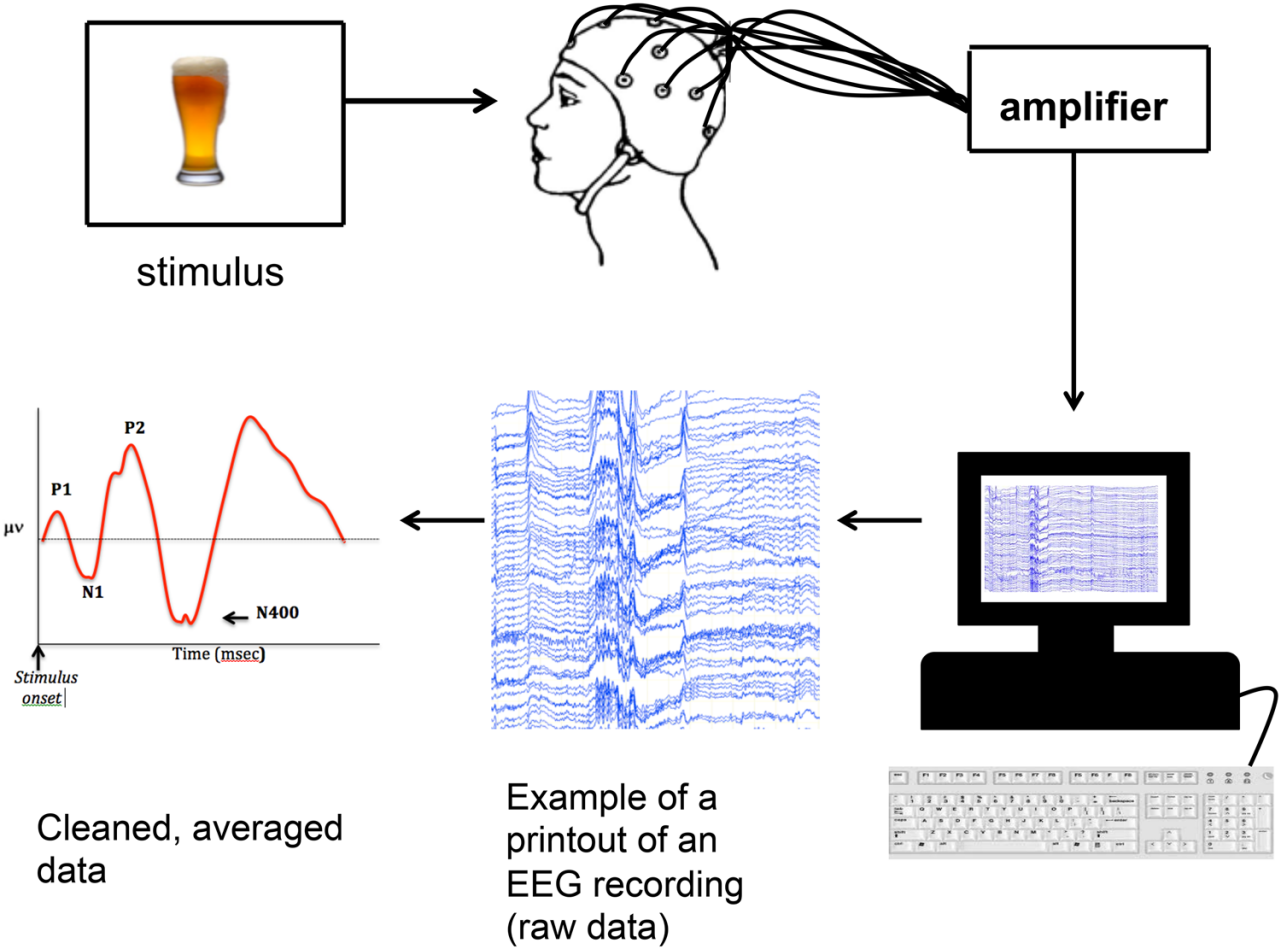
In an experimental setting, a number of electrodes, usually 32, 64, or 128, are placed on a participant’s head, which enables the electrical brain activity to be measured on the surface (scalp)

Below is an example of an experimental setting used in ERP studies (Fig. 1).

**Fig. 2:**
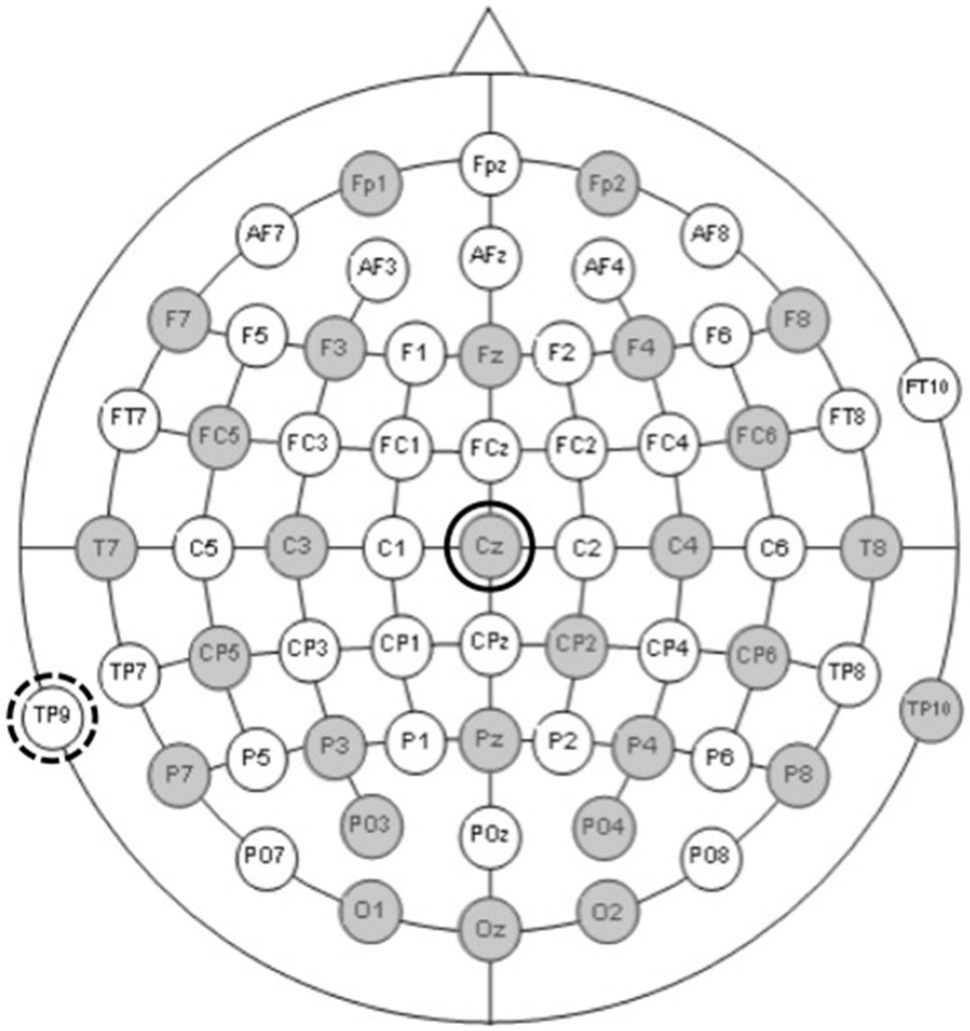
An example of the electrode array frequently used in EEG studies

The participant is presented, in this case, with a visual stimulus (a picture) on a computer screen. Each time it appears, it evokes a response by the brain, which is continuously recorded on the computer. This recording is then averaged, and individual ERPs, time-locked to the stimulus that was repeatedly presented, get extracted. EEG signal is obtained by recording the electrical activity, which is produced by the brain with the use of electrodes set at different sites on scalp (see Fig. 2, below, for an example of the electrode array that is often used in EEG studies, showing 64 sites, evenly distributed according to the 10/20 convention, with a Cz electrode as the reference). The EEG measures the difference in electrical potential between two sites (usually termed active and reference) measured over time. Eye-movements and blinks can contaminate the EEG recording and are “the result of the intrinsic voltage gradient of the eye” (Luck 2005, p. 162). The EEG recording can be visually inspected for eye-blinks as these are distributed across the scalp but have the most power in the frontal region, and inverse potentials are often found over the parietal and occipital sites.

### ERP Components

**Fig. 3:**
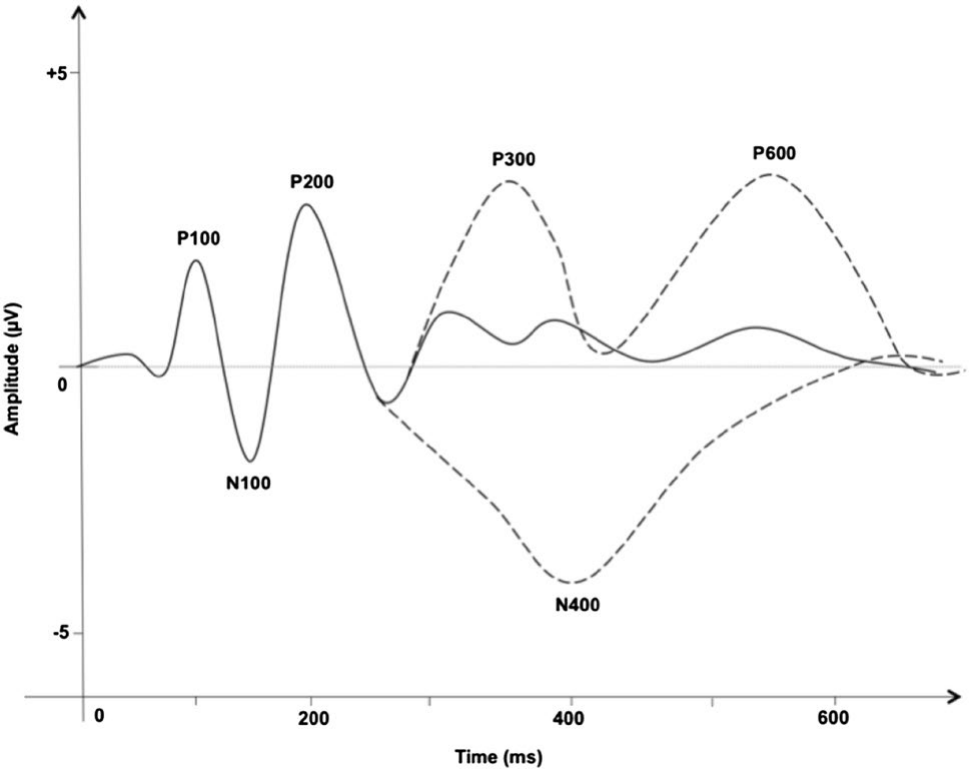
Some of the most commonly studied ERP components in language research. *Solid line* represents the basic perceptual components in an experiment involving any visual stimuli, such as words or pictures. *Two dashed lines* represent additional components, which are elicited in certain experimental designs

The point of extracting and analyzing ERP components from the continuous data in research is to average the activity over a number of trials in each condition. Only the activity that is recurrent and time-locked will not be cancelled out and therefore has some meaning. The output which is obtained, resembles a wave, with a number of positive and negative peaks. Those peaks are known as ‘components’ and labelled according to their polarity, with P standing for positive, and N for negative, and their approximate latency in milliseconds (for example, P100 is the positive peak appearing around 100 ms after the onset of the stimulus). Negative going waves are associated with activation, whereas positive going waves with inhibition. Medical experts, such as neurologists, usually read EEG with negative up and positive down. There is some inconsistency amongst the ERP researchers, with some following the neurologists’ convention and others having positive up (as illustrated in Fig. 3, below). There are many known ERP components involved in language research. Figure 3, below, presents some of the most studied ERP components.

Some, especially the early components (P100, N100, P200) are generally linked with basic, low-level perception and are thought to be automatic in nature. This means, that as long as a perceptual stimulus such as a word or a picture is presented, they should be elicited. Other components, which come later (usually after 250 ms.) represent conscious cognitive processing and can be elicited in certain experimental conditions. This distinction of early (automatic) and late (more conscious) components is made just to illustrate the general idea of ERP components. This generalization may not be valid in all cases, and the functional significance of each peak is often task-dependent and the experimental paradigm should always be considered.

### Components Most Commonly Implicated in Language Research

#### What Do You *Mean*? The N400

N400 is one of the best and most studied language components. It was first reported by Kutas and Hillyard (1980). Over the last 30 years, it has been used as a dependent variable in more than 1000 studies, with topics ranging from language comprehension, through semantic memory, processing of faces and gestures, to clinical studies such as those that looked at developmental disorders. It was discovered by accident, in a modified experiment aimed at eliciting a P3b response for language materials (Kutas and Hillyard 1980). Participants were presented word-by-word with sentences, which had either a congruent ending (75% of stimuli), such as ‘I shaved off my moustache and *beard*’, and 25% of sentenced which either had a strange ending (‘He planted string beans in his *car*’) or completely incorrect endings (‘I take my coffee with cream and *dog*’). As a result, a large negativity was elicited—largest for semantically anomalous sentences, but present also for those that had a strange, although theoretically possible, ending. It peaked around 400 ms after the onset of target word, and was labeled the N400 (Kutas and Federmeier 2011).

The N400 is found in a number of different experimental paradigms. Firstly, it has been well documented in lexical priming paradigms. The N400 effect (created by the different waves of the congruent—incongruent stimuli) is found when the target word is unrelated (for example, semantically or categorically) to the proceeding word (prime). The unrelated pairs induce larger N400 amplitudes as opposed to the related ones. Secondly, it has been found in auditory word presentation (Bentin et al. 1993). In those experiments, the N400 appears slightly earlier than in the case of visual word presentation (but only in natural speech; when presented at a fixed rate, there is no shift in timing) and lasts longer. It also has a more frontal topography, less concentrated on the right (Holcomb and Anderson 1993).

It is important to note that the N400 is not elicited to just any unexpected language manipulations. For example, it is not found in studies using capital letters for the target word when the rest of the sentence is in lower-cases (for example, ‘I shaved off my moustache and *BEARD* **’**) or those using simple grammatical violations, such as having a singular form of the noun when it should have been plural (for example, ‘All turtles have four *leg*’). The N400 effect is not elicited as a response to just any violation, linguistic or not, but rather is very closely linked with the processing of meaning. It is especially powerful in language studies, however its application goes beyond that (Kutas and Federmeier 2011). The component’s sensitivity to meaningful stimuli enabled a range of semantic investigations to take place, including those that focus on how the semantic memory is stored and retrieved in the brain (commonly referred to as semantic memory).

Kutas and Federmeier (2011) point out that out of a range of different studies, two main streams of findings are prevalent. Firstly, there is a disparity between the behavioral outcomes (reaction times) and the ERPs. That is, only rarely do those two behave in a similar pattern. This is perhaps not surprising given that with behavioral measures such as RTs, a number of cognitive processes take place and get consolidated by the time the individual makes a response; whereas with the ERPs, the components of interest reflect only a specific fraction of the whole process. Secondly, the results obtained through the ERP analysis often fail to fully support an existing theory, thus supporting different elements of various theoretical approaches.

The N400 has been reported across different modalities, such as speech production (Strijkers and Costa 2011) sign language (Kutas et al. 1987), and pseudowords (Leinonen et al. 2009; Friedrich et al. 2006), pictures (Nigam et al. 1992; West and Holcomb 2002; McPherson and Holcomb 1999), faces (Debruille et al. 1996; Barrett and Rugg 1989). However, it is important to mention that topographies of the N400 distributions can vary in each of those contexts (Kutas and Federmeier 2011). As the N400 effect has been found in studies that used different types of stimuli, such as words, pictures, and sounds, amongst others, Kutas and Federmeier (2011) argue that the N400 effect should be seen as “modality-dependent but not modality-specific” (p. 9). That is, different types of stimuli can elicit the N400s, but those, although they have many broad similarities (such as waveshape and timecourse), depend on the type of stimuli used and vary in terms of the specifics (especially when it comes to topography). And so, for example, written words elicit the N400 which is strongest in centro-parietal region, whereas pictures are concentrated in the fronto-central regions. Furthermore, when comparing within-participant differences, van Petten and Rheinfelder (1995) found that when presented with visual/auditory words and meaningful environmental sounds, either meaningfully related or not to the upcoming words, the N400 was more right hemisphere dominant in the case of words, but left hemisphere dominant in the case of environmental sounds. Even though the N400 has been found in studies using sounds, the N400 effect does not reliably appear in classic music experiments. Instead, those consistently elicit a P3b response (Besson and Macar 1987).

Looking at the wide range of studies that incorporate the N400 as a measurement, it is difficult not to conclude that our unique ability to see the *meaning* in the world around us, to which the N400 is susceptible, is underpinned by a number of cognitive processes, such as attention, memory, language, perception, amongst many others. The N400 therefore seems to be a very reliable and solid component with which we can study processes directly linked to semantic integration.

#### The Preceding Context Matters: The P600

P600 is a positive wave of activity occurring around 500–800 ms after the onset of a target word. It is an index of processing syntactically incorrect or non-preferred sentences (Osterhout and Holcomb 1992; Hagoort et al. 1993). Osterhout and colleagues (1994) argue that the P600 component varies with the degree to which a syntactic continuation of a sentence is expected. That is, grammatically incorrect continuations result in a larger P600 than those that are grammatical but non- preferred. It has also been found to be sensitive to continuations which are more difficult to process even though they are grammatically correct and preferred, compared to a control condition (Kaan et al. 2000). Additionally, recently the P600 has been linked with monitoring and re-evaluation processes (Kolk et al. 2003; Kolk and Chwilla 2007; van de Meerendonk et al. 2010). Therefore, the P600 can reflect the ‘double- checking’ of the information that is expected in the processing of syntactically abnormal sentences. In addition to syntax, the P600 has been found to be elicited in some situations outside of the language context, such as violations in music (Besson and Macar 1987; Patel et al. 1998), mathematics (Lelekov et al. 2000), and sequencing (Lelekov et al. 2000; Núñez-Pena and Honrubia-Serrano 2004). This implies that the P600 component is sensitive to a violation of any expected structure, whether linguistic or not.

Some investigators took advantage of the fact that some syntactic violations can impact semantics, and investigated the processing of less clear-cut psycholinguistic aspects, such as the gender agreement between a pronoun (her) and its antecedent (the boy). In this example, such processing could be argued to be syntactic (constrained by an individual’s grammar) or semantic (part of the word’s meaning and how those words are used in speech). The results of such experiments clearly show that similar violations only modulate the P600 (and possibly LAN) rather than the N400, suggesting that they were perceived as syntactic rather than lexico-semantic (Osterhout and Mobley 1995).

#### Left Anterior Negativity (LAN)

Another component, known to also index syntactic processing, is the left-anterior negativity (LAN). It is a negative wave of activity, peaking around 300–500 ms window on the frontal part of the left side of the scalp. However, this location has not been found to be consistent across studies (Hagoort et al. 2003). It is elicited by grammatical violations (Kutas and Hillyard 1983; Friederici et al. 1993) and garden path sentences (Kaan and Swaab 2003), albeit less frequently. A distinction can be made between an early LAN (ELAN), elicited around 100–200 ms after stimulus onset, and LAN appearing in the 300–500 ms window. The ELAN has been implicated in automatic processing of phrase structure information and is elicited when phrase structure or word category is violated, such as when a passive participle (not a noun) follows a determiner (for example, *the stolen car*) (Neville et al. 1991; Friederici et al. 1993). The later component, LAN, has been initially implied by Friederici and colleagues (2002) to be elicited by problems with morpho-syntactic agreement process, but later this interpretation has been questioned. Hagoort and colleagues (2003) elicited it for phrase structure violations, and Deutsch and Bentin (2001) found it to be an early index for agreement violations. There is a debate about the language specificity of the LAN, with some researchers claiming that it reflects processes specific to syntax, whilst others argue that it is a more general index of working memory load (Kluender and Kutas 1993a, b; Coulson et al. 1998).

In the literature, it is common to find (E)LAN component(s) followed by the P600, with the P600 thought to reflect post-hoc integration of various streams of information and the repair of anomalies involving sentence structure, and possible semantic inconsistencies.

#### Mismatch Negativity (MMN)

This component reflects auditory deviance, and therefore is used in speech perception research. It is a negative deflection, peaking around 160–220ms after stimulus onset (Luck 2005). It is elicited in an auditory oddball paradigm, in which one ‘standard’ sound is presented frequently, whist another, ‘deviant’ (which differs in pitch, duration, and other acoustic/phonetic properties from the standard) is presented randomly and infrequently. MMN is observed if the difference between the standards and deviants has been registered. MMN has been suggested to act at the pre-attentional level, since it can be elicited in coma individuals (Näätänen 2003). It can also be elicited when listening to music, watching a movie, reading a book, or sleeping, and individuals do not have to engage in a specific activity. MMN is believed to be a result of an automatic process, which compares actively incoming stimuli (sounds) to a sensory memory trace of previous sounds (Luck 2005). MMN has been used extensively in first language acquisition research with neonates and infants. Typically, behavioral research on speech perception in babies focuses on preferential looking or sucking rate (Vouloumanos et al. 2010; Werker et al. 1998). However, such methods are sometimes difficult to quantify and their interpretation has, at times, caused controversy (Cheour et al. 1998). An example of research using ERPs and the MMN is looking at how children’s perception of phonological categories changes with age and development. Initial behavioral reports indicated that until about 1 year of age, infants are sensitive to all kinds of phonemic distinctions, whether they are present in their native language or not (Werker and Tees 1994). Research involving ERPs has replicated those findings. In adults, a smaller MMN is elicited to vowel categories, which are not present in their native language (Näätänen et al. 1997). When comparing Finnish babies, they showed a larger MMN to /õ/ found in Estonian language at the age of 7 months, than at the age of 11 months (Cheour et al. 1998). Additionally, the MMN elicited by the 11 month old babies to the Estonian /õ/ was smaller than the MMN elicited by their peers for whom Estonian was a native language. Therefore, a conclusion can be drawn that infants become less sensitive to their non-native phonemic distinction as they grow. This example illustrates how ERPs can be used in language research with infants.

### Pros and Cons of Using ERPs in Language Research

#### Advantages

ERPs, because of their good temporal resolution, are useful in language research because language processing happens at a very fast pace. Individual words are recognized in much less than half a second, and a difference between a /*d*/ and a /*t*/ can be recognized in just a few milliseconds because it comes down to a difference in voicing onset (Kaan 2007). Only a method with an excellent temporal resolution can provide some insight into how language processing unfolds over time.

ERPs are particularly useful when working with clinical populations, such as individuals with aphasia, or infants and children. It is a technique that enables researchers to present the stimuli in spoken form, rather than in writing. Additionally, participants are not required to perform an extra task, again making it particularly useful when testing special populations. For example, in studies involving language comprehension, it is possible to assess the processing of a particular word, which might appear in the middle of the sentence. In classic behavioral experiments, it is necessary to wait until the sentence is fully presented for participants to make a response, thus relying on their memory. By that time, a number of cognitive processes are involved and it is not possible to accurately determine their effect. In experiments where an overt response by participants is needed, it is possible to establish which stage of processing is affected by a particular experimental manipulation, from the stimulus presentation to the response.

This technique allows for the collection of continuous data, with very good temporal resolution. In many language experiments, the sampling rate (defined as the rate at which the waveform data is sampled so that it can be changed to a numerical format) is between 250 and 512 Hz (samples every second), which is in line with a rate of language comprehension and therefore enables the continuous online processing.

The ERPs are multidimensional, allowing researchers to draw conclusions about the type of processes involved and their relationship. An example might include studies involving the RTs and self-paced reading. In those cases, we do not know why people might struggle with reading, whereas with the ERPs we can say whether they are struggling with, for example, the semantics or syntax, as those are different components responsible for different processes and they are easily distinguishable in the ERPs.

Another advantage is that the effects can be seen almost immediately upon presenting participants with a particular, for example semantic, manipulation. Additionally, every single stimulus, not just targets, can be time locked in ERP research, thus providing not only instantaneous, but also continuous look at language processing.

Finally, the cost of ERPs is important. ERPs are rather inexpensive, compared to other techniques. Whilst the cost of testing a single participant in the fMRI experiment was valued to be up to $800, the ERP session for each person was at $1–3 (Luck 2005).

#### Disadvantages

One of the main drawbacks of EEG research is the number of trials needed in an experiment. It is essential to have a large number of stimuli in each condition presented to participants, usually at least 40, because an individual ERP signal is such a small part of the continuous EEG recording that we need a larger sample size in a number of participants for meaningful interpretations. The exact number depends on a number of things, but typically in sentence processing studies involving 20 participants, a minimum of 40 trials is needed in each condition (Kaan 2007). This in itself can create a number of further problems. Firstly, the time it takes to prepare the stimuli. Stimuli used in experiments have to be closely matched on a number of characteristics, such as word length, familiarity, imageability, amongst many others. Creating a set of such stimuli will take time, especially given that it is advisable that no stimulus is presented more than once to the same participant. Instead, different sets of items are created, and these are counterbalanced across participants to ensure that all versions of the stimuli are presented the same amount of time throughout the experiment, but no participant is presented with the same version of a stimulus more than once. It can be very time consuming, especially in experiments that have a number of different conditions. Kaan (2007) suggests that in a study with four different conditions, it can take over a year to prepare a well-matched set of stimuli. Secondly, the large number of trials means longer experiments, especially if more conditions are needed, and that leads to tired participants. This in turn leads to a number of difficulties, such as possible alpha waves, participants paying less attention to the task, poor concentration, employing different processing and coping strategies, more missed trials, and so on. It is important to remember that poor quality data leads to more noise, which in turn leads to more trials that might need to be rejected. Another important aspect is the human factor. Participants can often get tired and fed-up with long, monotonous studies. That is why frequent breaks are advisable. It is thought that blocks lasting 5–7 min, separated by a short break, are optimal (Luck 2005). Thirdly, a large number of trials often lead to more artefacts and more trials that will have to be removed. Eye blinks, muscle movements, accidental head turns, even swallowing can create artefacts which will affect the ERPs. Some researchers choose to give specific instructions to participants, such as asking them to blink only at specified times, for example, after they have made a response and never during, whilst others choose to refrain from such specific instructions because it can affect participant’s attention and quickly lead them to be fatigued. A mathematical method, such as that proposed by Gratton and colleagues (1983) can be applied to the data in order to correct for the distortion of eye blinks, which can be especially useful in special populations, where specific instructions often cannot be given. However, using such method also has some drawbacks (Luck 2005). A large number of trials make it impractical for the ERPs to be used in certain experiments, such as those in which each participant can only receive one trial in each condition (Luck 2005).

Another possible disadvantage of using ERPs is the poor spatial resolution. ERPs show a good temporal resolution, but it is difficult to say where in the brain the activity occurs. Poor spatial resolution could be a result of limited spatial sampling or contamination of the reference electrode. Furthermore, ERPs only provide information about surface cortical sites, whereas other neuroimaging techniques, such as MRI, can go deeper in the cortex, or even subcortical, when looking at the activation patterns.

In sentence processing experiments involving visual presentation of stimuli, it is not advisable to present the whole sentence on the screen at once because of the eye movements. Also, because it would not be possible to time lock the ERPs to our target stimuli, sentences are usually presented on a word-by-word basis. This leads to a relatively slow stimuli presentation rate, usually of 500 ms interval from the presentation of one stimulus to the presentation of another. This is different from typical reading and can therefore add some load to working memory and may introduce a confound (Kaan 2007). However, results from experiments investigating natural speech are comparable with those using visual presentation of stimuli (Gratton et al. 1983).

In comparison with classic behavioral studies, the interpretation of ERPs is less clear and requires much more inferences. For example, in behavioral experiment in which participants took 30 ms longer to press the button in condition 1 than in condition 2, it is reasonable to say that the time to process and perform an action takes this much longer in condition 1 than 2. However, in ERPs when peak latency is later in condition 1 in comparison to condition 2, it is difficult to draw a conclusion without making many assumptions and inferences (Luck 2005).

Despite those possible limitations, using ERPs in language research has proven to be popular and valuable and has added a great depth of knowledge to how language is processed in the human brain in real time.

## Conclusions

Although not without some limitations, EEG has been successfully used in cognitive psychology research. In particular, thanks to its ability to directly measure real-time brain activity, especially without the need for an additional task, it has been extensively applied in the language domain. It is clear that ERPs can highlight the temporal unfolding of neural activity associated with different cognitive aspects of language comprehension and production.
